# Acupuncture for the treatment of thalamencephalic and mesencephalic injury secondary to electrical trauma: A case report

**DOI:** 10.3389/fnins.2023.1139537

**Published:** 2023-03-06

**Authors:** Hailun Jiang, Yi Zhang, Jieying Zhang, Boxuan Li, Weiming Zhu, Chaoda Liu, Shizhe Deng, Yuzheng Du, Zhihong Meng

**Affiliations:** ^1^National Clinical Research Center for Chinese Medicine Acupuncture and Moxibustion, Tianjin, China; ^2^Tianjin University of Traditional Chinese Medicine, Tianjin, China; ^3^First Teaching Hospital of Tianjin University of Traditional Chinese Medicine, Tianjin, China

**Keywords:** acupuncture, central nervous system disease, brain injury, neurological sequelae, electrical trauma, cerebral infarction

## Abstract

In a case of thalamencephalic and mesencephalic injury secondary to electrical trauma, a 29-year-old patient has been receiving manual acupuncture for 17 months in National Clinical Research Center for Chinese Medicine Acupuncture and Moxibustion. As a result of treatment, the patient's self-care ability and quality of life have greatly improved. In order to fully understand how acupuncture can benefit neurological sequelae resulting from electrical trauma, further research is needed. Additionally, there should be consideration given to the promotion of acupuncture therapy in the neurological sequelae of electric shock.

## Introduction

An electric injury is caused by artificial current flowing through the body. Patients are more likely to be male, and the majority of these injuries are caused by low voltage (<1,000 V) accidents or work-related accidents. The burn ward or intensive care unit (ICU) is typically the first place a patient is admitted due to severe systemic burns after an electrical injury. However, due to the diversity of its clinical manifestations involving multiple organs and systems, it is often requires the cross-pollination of multiple disciplines for unconventional diagnosis and treatment, moreover, the prevention, follow-up, and treatment of complications following electrical trauma are vital.

When the current penetrates irregularly into the deep tissue of the body, aside from the appearance of clear boundary burning on the skin (redness, blisters, breakage, etc.), it can also damage the nervous system directly. There are several types of nerve injuries, including ischemic encephalopathy, cerebellar ataxia, hypoxic encephalopathy, intracranial hemorrhage, damage to other areas of the central nervous system, and peripheral nerve injuries. In electrical shocks, nerve tissue has a low electrical resistance compared to other tissues, explaining the high incidence (31~81.6%) of nervous system injury (Singerman et al., 2008; Warenits et al., 2020). Furthermore, owing to the gap between developing and developed countries in health care construction and the results of epidemiological investigations of electrical injuries reported in different regions are inherently different, it is possible that the true incidence of nervous system injury after electrical shock is higher in developing countries and higher than the figures reported above.

According to current literature, neurologic syndromes after electrical injury can be classified in two classifications. Cherington' classification: Cherington (2003, 2005) proposed four groups of electrical injury based on time of onset, duration of symptoms, and severity of the clinical situation and whether a secondary event to other mishaps (I. immediate and transient, II. immediate and prolonged or permanent, III. delayed, IV. traumatic lesions secondary to falls and blast effects). Andrews' classification: Andrews and Reisner (2017) framed the electrical injury in an anatomic classification (I. Central syndromes, II. Cerebellar syndromes, III. Cranial nerve and related disorders, IV. Autonomic syndromes, V. Spinal cord disorders, VI. Paralyzes, VII. Peripheral nerve disorders, VIII. Sensory abnormalities). Understanding the damage to the nervous system after electrical trauma is crucial for developing effective treatment plans to improve quality of life for survivors. In spite of the fact that neurological sequelae are very common after electric injury, there are no guidelines or reports that give a clear treatment plan for acute ischemic stroke after electric shock, which is one of the most severe neurological complications.

Acupuncture is one of the traditional Chinese medicine treatments, which is a therapeutic operation *via* nondrug afferent stimuli. As a representative of the external treatment of traditional Chinese medicine, acupuncture has been inherited through the generations and still plays an important role in clinical treatment. The research results of acupuncture have been published many times in top international journals such as Nature, BMJ, JAMA and Annals of Internal Medicine, indicating that the effectiveness of acupuncture therapy is gradually gaining international recognition (Liu, 2022). The disease spectrum of acupuncture has been continuously expanded, especially in nervous system diseases, and its safety and effectiveness have been continuously confirmed by modern evidence-based medicine (Xu et al., 2018; Lu et al., 2022). In China, acupuncture treatment for ischemic strokes has been included in traditional Chinese medicine (TCM) rehabilitation guidelines. There are a variety of regulating effects of acupuncture on the nervous system, which are capable of promoting the healing of various injuries of the nervous system and improving neurological function problems. Hence, it is recommended as a level A evidence in the TCM rehabilitation guidelines for the treatment of ischemic stroke and as a level I evidence (Zhang et al., 2021). Considering this, we came up with an interesting question—could acupuncture be used to treat neurological sequelae caused by electrical trauma? However, only four cases of acupuncture as a treatment for electrical injury-induced neurological sequelae have been reported to date and are all reported in China (Huang, 1957; Zheng, 1986; Shao, 1997; Hao, 2005). Despite electrical current is known to cause damage to the nervous system, the pathogenesis of nerve injury after electrical trauma is unknown. The evidence that acupuncture can treat various nervous system diseases after electrical trauma has not been fully explored.

We report a case of thalamencephalic and mesencephalic injury secondary to electrical trauma. By Cherington's classification, he was categorized as a group II, and By Andrew's classification, he showed symptoms of types I and III. In the second month after electrical trauma, the National Institute of Health stroke scale (NIHSS) score was significantly reduced after 3 weeks of acupuncture treatment (Score dropped from 9 to 6, and language function improved). The patient was able to walk independently after 17 months of continuous acupuncture treatment (the NIHSS score had reduced to 3, and motor function improved). There has been a significant improvement in quality of life. We hope to share this case report with colleagues around the world *via* communication platforms.

## Case description

In April 2021, a 29-year-old right-handed man suffered an electrical injury after coming into contact with a 220 v wire in the staff dormitory. When a colleague discovered the condition, the patient was unconscious and had urinary incontinence. Then, the patient was rushed to the hospital's emergency room. At that time, the patient had partial exfoliation of the epidermis of both great toes, as well as subcutaneous congestion around the orbit of the left eye and left shoulder. After analeptic and diuretic treatment, the patient was transferred to the burn department for indwelling gastric tube and urinary tube, and received a higher level of care. Additionally, neurological physicians and neurosurgeons was consulted immediately.

**Figure 1:**
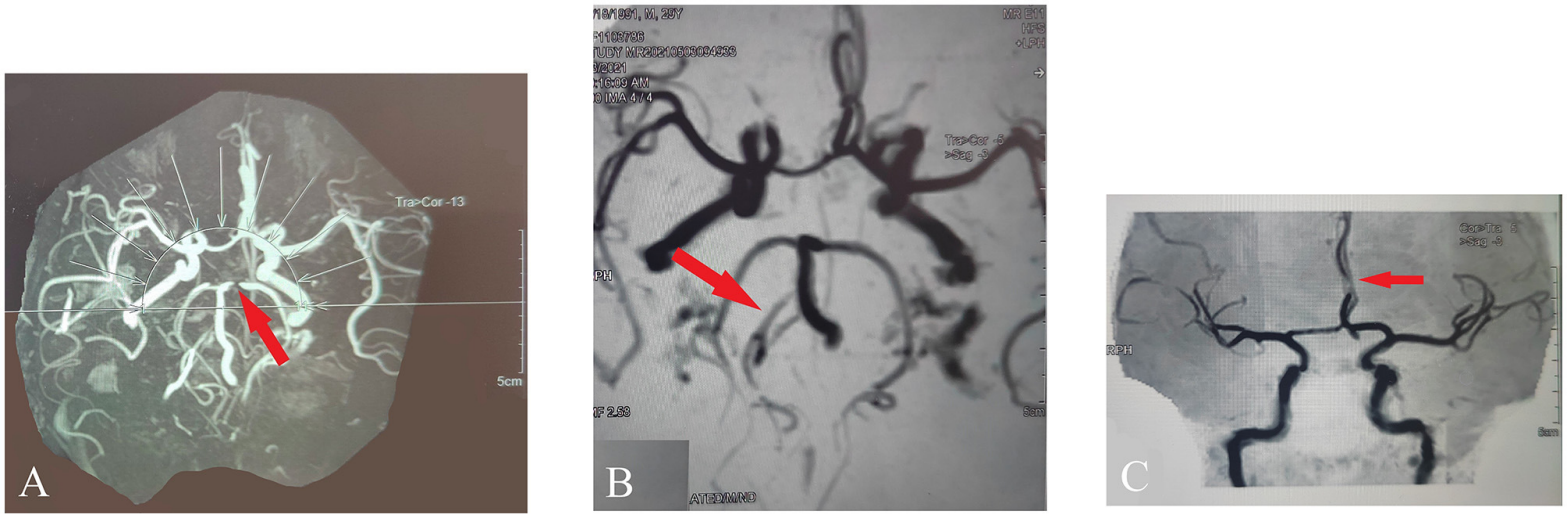
MRA revealed the artery's condition. **(A)** Localized stenosis of P1 segment of bilateral posterior cerebral arteries. **(B)** Slender right vertebral artery. **(C)** Localized stenosis in the A2 segment of the bilateral anterior cerebral artery.

Initially, the patient was in a coma with bilateral pupils of equal size and roundness, both 8 mm in diameter, loss of light reflex, and positive Babinski sign. On admission, magnetic resonance imaging (MRI) of the cervical spine showed that the spinal cord measured normal in size, shape, and signal, the cervical disc herniation compressed the dural sac, the left vertebral artery's flow void signal was decreased, and the right vertebral artery was thin; magnetic resonance angiography (MRA) revealed the localized stenosis of P1 segment of bilateral posterior cerebral arteries (panel A), a poor local visualization of the C5 segment of bilateral internal carotid arteries, and the basal artery ring to be normal in size and shape, as shown in Figure 1; MRI of the head showed abnormal signals in midbrain and bilateral thalamus, considered cerebral infarction (top of the basilar syndrome), and soft tissue swelling was observed in the bilateral frontal, left parietal temporal, and left orbit areas. The patient became incapable of spontaneously expectorating 2 days after admission to the hospital, so a tracheotomy was performed. After examining the patient's neurological system, it was recommended that the patient repeat the MRI. A repeat imaging performed 10 days after the original MRI showed abnormal signals in the bilateral thalamic region, and midbrain area. MRA of the head showed localized stenosis in the clinoid segment of the bilateral internal carotid artery, slender right vertebral artery (panel B), and localized stenosis in the A2 segment of the bilateral anterior cerebral artery (panel C), as shown in Figure 1. As the patient was being hospitalized, his state of consciousness changed from coma to drowsiness, and he was able to cooperate with simple commands (See Supplementary Video 1). A 41-day period after the electrical injury, the muscle strength of the patient's limbs recovered to grade 3. Approximately 70 days after the shock, the patient came to National Clinical Research Center for Chinese Medicine Acupuncture and Moxibustion for acupuncture treatment in hopes of improving his quality of life and self-care abilities.

**Figure 2:**
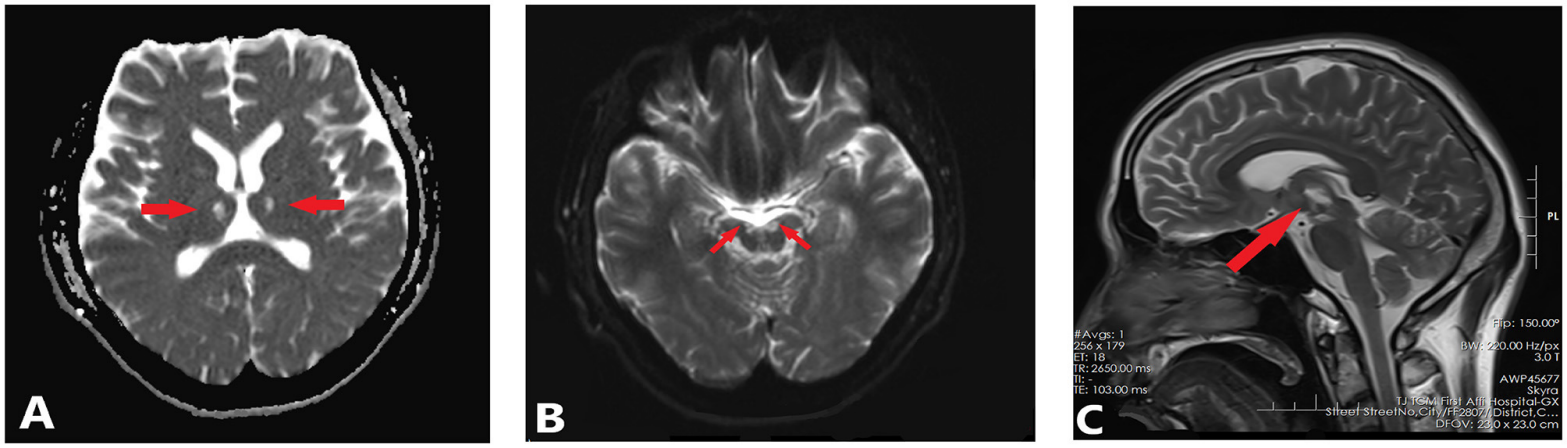
MRI revealed abnormal signals in the midbrain and thalamus. **(A)** The axial scanning of MRI revealed bilateral thalamic infarcts (DWI, diffusion-weighted imaging). **(B)** The axial scanning of MRI revealed Mesencephalic infarction (DWI, diffusion-weighted imaging). **(C)** The sagittal scanning of MRI revealed Mesencephalic infarction(T2-weighted MR images).

At the time of his arrival at center's inpatient ward, the patient had a NIHSS score of 9 (2 points for the level of consciousness question and 2 points each for the upper and lower extremities movements, 3 points for language), dilated and stiff pupils. After 24 days of hospitalization, the patient was discharged with a NIHSS score of 6 (2 points each for the upper and lower extremities movements, 1 points for language, and 1 point for dysarthria) and there was no significant change in the pupil condition, eye movement showed external rotation, but no internal rotation, up rotation, or down rotation was demonstrated, and both right and left eyelid levator muscles were grade 0 in strength. In Figure 2, abnormal signals are still visible in the thalamus and midbrain after the MRI scan taken on December 28, 2021. As part of his rehabilitation treatment, the patient received regular acupuncture sessions 5–10 times per week in center's outpatient department and ward.

As for 5 December 2022, the patient had been receiving acupuncture for more than 17 months. Now he is able to walk without the cane, regain language function, and carry out daily communication with people independently, which has greatly improved his quality of life, but it is still difficult to lift the upper eyelid with right and left eyelid levator muscles due to a lack of strength in these muscles. In Supplementary Videos 2–4, you can see the effect of acupuncture on the speech and motor skills of the patient.

## Acupuncture treatment

**Figure 3:**
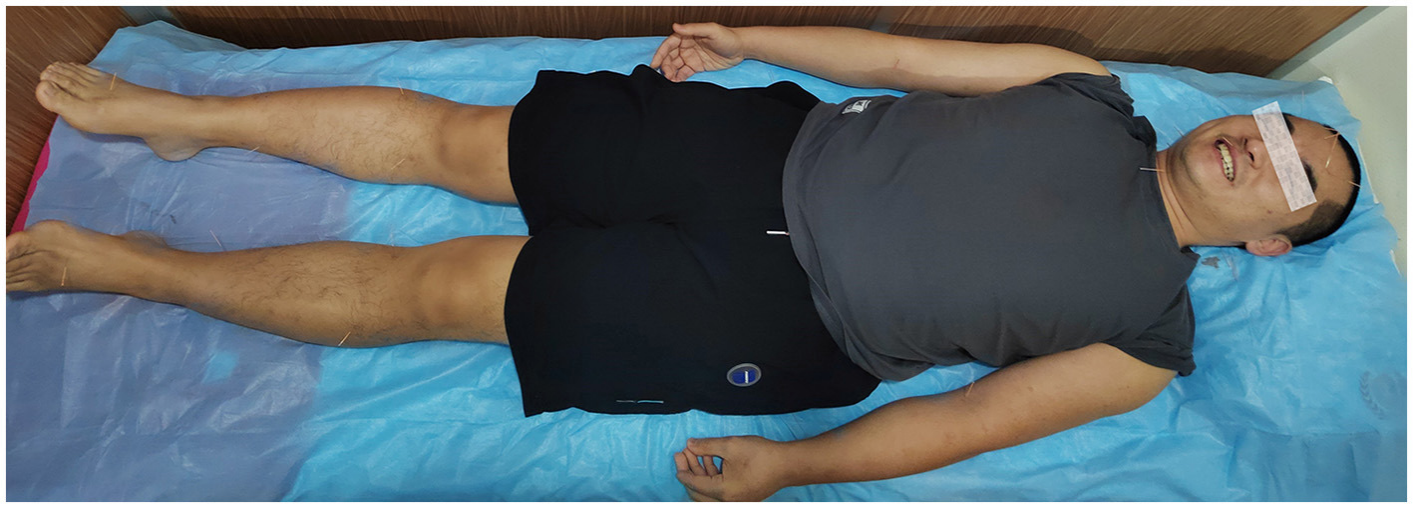
The selected acupoints.

An acupuncturist with 20 years of experience in acupuncture provided him with acupuncture treatment based on the principle of “XingNaoKaiQiao.” Patients are treated with matching acupoints based on their specific symptoms, such as such as limb weakness and language dysfunction, depending on the degree of their symptoms at each visit. As the patient lay supine, disposable filiform needles were inserted into the acupoints after skin disinfection. After manipulation, the needle was retained for 30 min each time. Table 1 shows the commonly used acupoints and their location, along with acupuncture manipulations. Figure 3 illustrates the acupoints for acupuncture.

**Table 1:** Characteristics of acupuncture.

**XingNaoKaiQiao acupuncture therapy**	**Acupoint selection**	**Acupoint location**	**Acupuncture manipulation**
Mainpoints	Neiguan (Bilateral PC6)	In the volar aspect of the forearm, 2 cun above the wrist crease, between the tendons of radial wrist flexor and palmaris longus.	Puncture perpendicularly for 0.5–1 cun, using combinative reducing method (lifting-thrusting and twirling-rotating) for 1 min.
	Shuigou (Unilateral GV26)	In the face, at the junction of the upper 1/3 and middle 1/3 of the philtrum groovet.	Puncture obliquely upwards to the nasal septum for 0.3−0.5 cun with heavy bird-pecking method until the patien's eyeballs are moistened or tears flow.
	Sanyinjiao (SP6, bilateral)	In crus inside, 3 cun above the tip of the medial malleolus, at the posterior border of the tibia.	Puncture obliquely for 1~1.5 cun, at the angle of 45° with the skin surface along the posterior border of the medial aspect of the tibia, with heavy insertion and light lifting manipulation to make the affected leg twitch three times.
Auxiliary points	Xiajiquan (Lower HT1, bilateral)	At the inner side of the upper arm, 1~2 cun down from HT1, avoid axillary hair, on the muscles.	Puncture perpendicularly for 1~1.5 cun with light insertion and heavy lifting manipulation to make the affected arm twitch three times.
	Chize (LU5, bilateral)	In the elbow, at the mid point of cubital crease, and the radialis depression of the biceps tendon.	Puncture perpendicularly for 1 cun, then lift and thrust needle until the needling feeling radiates from the elbow joint to the fingers.
	Weizhong (BL40, bilateral)	In the posterior region of knee, at the midpoint of popliteal crease.	The doctor lift the affected leg with one hand and against the knee joint with the elbow in order to straighten the affected limb and fully expose the popliteal space. Take the needle with the other hand, puncture perpendicularly or obliquely with the needle tip outward for 1~1.5 cun, with light insertion and heavy lifting manipulation to make the affected leg twitch three times.
	Lian Quan (RN23, unilateral)	Above the prominentia laryngea, at the depression of superior border of hyoid bone.	Puncture perpendicularly for 1 cun, then twirling manipulation with the frequency of 60 times/min for 1 min.
	Yintang (EX-HN3, unilateral)	On the forehead, the midpoint between the glabella.	Puncture horizontally for 0.3~0.5 cun with light bird-pecking method
	Fengchi (GB20, bilateral)	In the posterior cervical region, below the occipital bone, in the depression between the superior end of the sternocleidomastoid and the superior end of the trapezius.	Puncture perpendicularly for 1~1.5 cun, then twirling manipulation with the frequency of 60 times/min for 1 min.
	Wangu (GB12, bilateral)	In the head, in the posteroinferior depression of the retroauricular mastoid.	Puncture perpendicularly for 1~1.5 cun, then twirling manipulation with the frequency of 60 times/min for 1 min.
	Tianzhu (BL10, bilateral)	In the nape, in the hairline depression of the outer edge of the trapezius.	Puncture perpendicularly for 1~1.5 cun, then twirling manipulation with the frequency of 60 times/min for 1 min.
	Zusanli (ST36, bilateral)	On the anterolateral side of the calf, when the lateral knee is 3 inches below, 1cun beyond the anterior border of the tibia.	Puncture perpendicularly for 1.5 cun without manipulation
	Taichong (LR3, bilateral)	On the dorsal side of the foot, in the depression preceding the junction of the first and second metatarsal bones.	Puncture perpendicularly for 1 cun without manipulation
	Yinlingquan (SP9, bilateral)	In the depression of the lower margin of the medial tibial condyle.	Puncture perpendicularly for 1.5~2 cun without manipulation
	Yangbai (GB14, bilateral)	On the forehead, just above the pupil, 1 cun above the eyebrow.	Puncture horizontally for 0.3~0.5 cun without manipulation

## Discussion

When an electrical accident occurs, the severity of tissue damage is closely related to characteristics of the power supply (low voltage <1,000 V or high voltage >1,000 V), the current type (alternating current or direct current), the propagation path of the current (Through chest or not), and the duration of contact (Bailey et al., 2007; Ahmed et al., 2021). Unlike lightning or direct current, patients always were exposed to alternating current for longer periods of time. There is a tendency for electric currents of 50 Hz to cause tonic muscle spasms [the “not let go” reaction (Rådman et al., 2016)], making the patient unable to release the power source without the assistance of someone else.

Annually, more than 3,000 people are admitted to burn centers in the United States because of electrical burns (Spies and Trohman, 2006). Burns caused by electric shock account for <5% of all burn center admissions, but they are associated with significant morbidity and mortality (Shih et al., 2017). Worldwide, nerve injuries caused by low-voltage electricity are probably much more common than we realize. Many patients do not report or consult physicians in the first moments after an electrical injury because most neurologic symptoms are mild and have a delayed onset. According to case reports, a 3-year-old child developed lower motor neuron (LMN) facial nerve palsy 1 day after experiencing a low-voltage electrical shock, and a 72-year-old man developed progressive weakness in both upper limbs 9 months after receiving a high-voltage electric shock, however, no one initially believed electric shock had been involved (Tashiro et al., 2000; Reddy et al., 2020). Besides consultation delay, missed diagnosis is also one of the reasons for the small number of nerve injury reports after electric shocks. In most cases, electrical damage to tissue cannot be detected by visual inspection or physical examination, and its sequelae are often apparent only after a certain period of time. Due to this, there is a high risk that nerve fiber damage will be missed when the overall burn appearance is not severe. An animal study has shown that electric shocks most damage myelinated nerve fibers that are large and fast-conducting (Abramov et al., 1996). Electron microscopy has also shown significant myelinated nerve fiber degeneration in peripheral nerves exposed to low-voltage electric shocks (Yvon et al., 2018). There is persistent severe neurological impairment in 50–73% of patients with low-voltage electrical injuries according to follow-up studies (Grube et al., 1990; Hussmann et al., 1995; Singerman et al., 2008; Tamam et al., 2013). Thus it can be seen that an electric shock-induced nerve injury is characterized by high incidence, delayed onset, and concealment. Therefore, it is necessary to conduct nerve sensory tests in patients with electrical injury in time to verify whether there is potential damage to the nervous system. Furthermore, T1 and T2 weighted images of MRI can assess the specificity of brain tissue as well as neural parenchymal changes in the brain (Chandrasekhar et al., 2018), and diffusion tensor imaging (DTI) is an effective technical tool to evaluate nerve tract damage (Devale et al., 2017). Unfortunately, the patient in our case report did not undergo DTI. Despite the fact that most neurological deficits can be reversed, a small number of progressive and permanent deficits pose the greatest risk of disability. As we all know, “prevention” is more important than “treatment,” in addition to improving awareness of safe electricity use in daily life and enhancing emergency power outage measures, there is much more meaning in early detection of nerve damage symptoms and early application of neurotrophic and anti-inflammatory agents than in taking no measures to intervene the delayed symptoms.

In the published literatures, we found a case report of ischemic stroke after electrical injury in 2010. We compare the two cases in Table 2. In the 2010 case report, a patient underwent a low-voltage alternating current shock lasting 5 min and denied any head contusion, but developed an acute infarction in the right frontotemporal area involving the right basal ganglia and corona radiata. It is speculated by the authors of the 2010 case report that the ischemic stroke was caused by vasospasm or embolism of the supplying brain (Huan-Jui et al., 2010). We also thought that there is a high possibility of infarction of the apex of the brain stem caused by vasospasm due to the fact that our patient had no history of epilepsy or heart disease, and there was a similar electric shock experience with the above case (Unwitnessed continuous alternating current shock), in addition, there is radiographic evidence of vascular stenosis (The right vertebral artery is thin, the flow voids signal in the left vertebral artery has decreased, local stenosis of the P1 segment of bilateral posterior cerebral arteries, the C5 segment of bilateral internal carotid artery was poorly visualized). Further, the patient in 2021 case report has aphasia, cerebellar ataxia, visual field defects, oculomotor nerve palsy, and other manifestations of top of the basilar syndrome (Caplan, 1980). Of course, the presence of vasospasm needs to be confirmed further by transcranial Doppler (TCD). Based on the case report from 2010, it was necessary to use vascular spasmolytics (intravascular injection of nimodipine) and microcatheters to treat vasospasm and stenosis in the acute stage of electric injury, and at follow-up, the patient's dysarthria had improved after professional rehabilitation, but daily walking did not get rid of the quadricane (Huan-Jui et al., 2010). As a result of this case report, the patient's speech function improved almost perfectly without language training, and he did not need to use a walker to perform daily activities. A recent follow-up showed that the patient had recovered well, his weakness had significantly improved, and he can now live on his own at home. He described his condition as being between 70 and 80% recovered in his own words. Therefore, it is acceptable to include acupuncture as part of the rehabilitation treatment for patients who have suffered electric injuries to their nervous system. Acupuncture is widely used for treating neurological disorders, and has the advantages of being easy to operate, having few side effects, being safe, and so on. In response to questions about his current medication, the patient revealed that he does not currently take any medication orally due to potential side effects, which also indirectly proves that acupuncture is easily accepted by patients as a green treatment.

**Table 2:** Two cases are compared based on their characteristics.

	**Case in 2010**	**Case in 2021**
Age	50	29
Gender	Male	Male
Type of current	60-Hz, 110-V alternating current supply	50-Hz, 220-V alternating current supply
Previous health status	Without any systemic disease or drug history	Rhinitis (In addition: stroke history was nonexistent)
Duration of current	More than 5 min	Unknown
head contusion	Denied any head contusion happened during fall down	No history of falling
laboratory investigations	All results within the normal limits	C-reactive protein, alanine aminotransferase, leukocytes, neutrophils, myoglobin, creatine kinase, and creatine kinase isoenzyme all increased
electrocardiogram	Normal	Normal
symptom	Retain consciousness, left limb weakness, glossolalia	Loss of consciousness, urinary incontinence
MRI	An acute infarction in the right frontotemporal area involving the right basal ganglia and corona radiata	Bilateral thalamic and midbrain infarction
MRA	Segmental narrowing of the siphon of the right internal carotid artery (ICA) and the M1 segment of right middle cerebral artery (MCA)	The left vertebral artery's flow void signal was decreased, and the right vertebral artery was thin, the localized stenosis of P1 segment of bilateral posterior cerebral arteries, a poor local visualization of the C5 segment of bilateral internal carotid arteries, localized stenosis in the clinoid segment of the bilateral internal carotid artery, localized stenosis in the A2 segment of the bilateral anterior cerebral artery, and slender right vertebral artery.
Endovascular treatment in the acute phase	Intravascular injection of nimodipine and microcatheter manipulation	Intravenous drip nalmefene hydrochloride injection, Xingnaojing injection, glycerol fructose and sodium chloride injection, furosemide injection
rehabilitation program	Physical, occupational and speech therapy	Acupuncture
Remaining symptoms during follow-up	This patient can walk with quadricane for daily activities and dysarthria improved in 2 months.	19 months after electrical trauma: weakness of the left and right levator palpebrae superioris, almost perfect speech and freedom from crutches, but emotional abnormalities

It was recommended to the world in 1979 by the World Health Organization that 43 indications of acupuncture be used, including those related to neuromuscular conditions, and acupuncture was approved for clinical use during stroke rehabilitation by the National Institutes of Health in 1997 (Liu, 2022). Study has shown that acupuncture has a positive effect on the recovery of cerebral perfusion, reducing degree of neurological damage, and improving prognosis for neurological function after stroke, which may related to the fact that acupuncture promotes blood vessel regeneration through the regulation of hemodynamics and the release of vasoactive substances (Wang et al., 2022). Moreover, acupuncture therapy promotes synaptic plasticity and facilitates nerve repair through different ways (Qing et al., 2016). According to some studies, acupuncture has also been shown to be effective in regulating vasomotor contraction and preventing cerebral vasospasm (Ko et al., 2013; Li et al., 2022). However, it is unfortunate that the case report falls under Level IIIb of the evidence scale developed by the Center for Evidence-based Medicine at the University of Oxford and there are no systematic reviews or randomized controlled trials (RCTs) reports on acupuncture treatment for nerve sequelae resulting from electric injuries as far as. As a result, there is not enough evidence to support the use of acupuncture to treat nerve sequelae after electrical injury, and it is still difficult to promote its application. In spite of this, by sharing this case, we hope to inform colleagues that the use of acupuncture as an effective method in the management of neurological sequelae resulting from electrical trauma deserves further investigation.

Need to add that, as part of our communication with the patient and his family, the family mentioned that the patient often cried without reason, had a low mood, and was experiencing a certain degree of cognitive decline. But there were no descriptions or treatments for this in his medical or medication history. Review of the studies found that survivors often suffer from psychological problems after electric shock, and long-term neuropsychological effects are common, mainly including post-traumatic stress disorder (PTSD), depression, insomnia, anxiety, cognitive impairment, and personality changes (Haq et al., 2008; Ramati et al., 2009; Andrews and Reisner, 2017; Fadhilah and Amin, 2019). There is a possibility that the mechanism involves the excessive release of NO, cortisol, oxygen free radicals, and glutamate by current stimulation on a large scale, moreover, after stroke, calcium overload, inflammation, oxidative stress, and other cascade reactions will occur in the local brain tissue as a result of ischemia and hypoxia. Upon complex interactions, these pathological factors or links produce cytotoxic effects on the vascular wall and neuronal tissue, ultimately leading to the emergence of neuropsychological symptoms (Andrews and Reisner, 2017; Rubino et al., 2022). Hence, it is important to pay attention to the mental state of electric shocks survivors. There are already some evidence-based evidence that acupuncture can alleviate post-stroke depression and cognitive impairment after stroke (Zhang et al., 2010; Zhou et al., 2020; Xie et al., 2022). However, due to the lack of attention and records on the psychological symptoms of patients in the early stage, it is difficult for us to make a corresponding assessment of whether acupuncture can have a positive effect on the psychological state of patients at this moment. In future clinical treatments, we will continue to track anxiety and depression levels and cognitive ability of patient with electric shock injury, and use neuropsychological scales to measure whether acupuncture can improve their mental state and cognitive abilities.

## Conclusion

A variety of mechanisms may contribute to ischemic stroke caused by an electrical injury, one of which may be severe muscle spasm and sympathetic nerve excitation, resulting in vascular wall damage leading to vasospasm and thrombosis or embolism, another may be direct potential damage to the nervous system caused by the current. For doctors and electrical trauma patients, early professional neurological function tests should be performed to detect potential signs of neurological injury. Moreover, patients who have experienced electrical trauma may present with abnormal mental states, and managing PTSD and complex electrical trauma can be challenging for clinicians. Therefore, clinicians should pay extra attention to this issue and provide timely intervention. As shown in this case report, acupuncture combined with rehabilitation has a positive effect on the recovery from severe neurological injuries after electric shock, and is an effective, economical and safe auxiliary treatment.

## Data availability statement

The original contributions presented in the study are included in the article/Supplementary material, further inquiries can be directed to the corresponding authors.

## Ethics statement

Ethical review and approval was not required for the study on human participants in accordance with the local legislation and institutional requirements. The patients/participants provided their written informed consent to participate in this study. Written informed consent was obtained from the individual(s) for the publication of any potentially identifiable images or data included in this article.

## Author contributions

YD and ZM were responsible for the entire manuscript. HJ, YZ, and JZ conceived, designed, wrote, and revised the manuscript. BL, WZ, CL, and SD revised the manuscript and discussed interpretation. All authors approve the final manuscript prior to submission.
